# The bioinformatics of the finding that the hepatitis delta virus RNA editing mechanism by a conformational switch exists in genotype 7 in addition to genotype 3

**DOI:** 10.1093/bib/bbaf451

**Published:** 2025-09-03

**Authors:** Rami Zakh, Alexander Churkin, Marina Parr, Tamir Tuller, Ohad Etzion, Harel Dahari, Danny Barash

**Affiliations:** Department of Computer Science, Ben-Gurion University of the Negev, David Ben-Gurion Blvd. 1, Beer-Sheva 8410501, Israel; Department of Software Engineering, Sami Shamoon College of Engineering, 56 Bialik St. Be'er Sheva 8410802, Israel; Department of Software Engineering, Sami Shamoon College of Engineering, 56 Bialik St. Be'er Sheva 8410802, Israel; Department of Bioinformatics, Wissenschaftszentrum Weihenstephan, Technische Universität München, Maximus-von-Imhof-Forum 3, 85354 Freising, Germany; Department of Biomedical Engineering, Tel-Aviv University, Chaim Levanon Street 30, Tel-Aviv 6997801, Israel; Soroka University Medical Center, Ben-Gurion University, 151 Yitzhak I. Rager Boulevard, Beer-Sheva 8410501, Israel; Stritch School of Medicine, Loyola University Chicago, 2160 South First Avenue, Maywood, IL 60153, United States; Department of Computer Science, Ben-Gurion University of the Negev, David Ben-Gurion Blvd. 1, Beer-Sheva 8410501, Israel

**Keywords:** hepatitis delta virus genotypes, HDV database, RNA folding prediction, RNA editing, RNA conformational switching

## Abstract

Hepatitis delta virus (HDV) is geographically classified according to eight known genotypes. The combined hepatitis B-hepatitis D (HEPB-HEPD) disease is the severest form of chronic viral hepatitis in humans and is characterized by mortality rates of ~20%. Hepatitis delta virus has no FDA approved therapy and its only available vaccine is the one for HEPB. Because it is the smallest RNA virus known to infect humans, RNA folding predictions by energy minimization of the whole genome can reveal important information on functional RNA secondary structure elements within the genome. A public HDV database (HDVdb) contains 512 HDV strains on which various bioinformatics methods can be applied, aiming to detect strains that could perform RNA editing via conformational switching. Up to date, only one such strain from HDVdb was known to perform that, in HDV genotype 3. Our goal was to locate more such strains, both in genotype 3 and in other possible HDV genotypes. In past work, by an eigenvalue mathematical analysis, we made an initial prediction that this peculiar RNA editing mechanism also exists in HDV genotype 7. We hereby extend our earlier findings and present newly discovered HDV strains from multiple genotypes for further analysis of RNA editing sites within the virus. The relevant strains taken from HDVdb are from both genotype 3 of Peru and genotype 7 of Cameroon. Additionally, the new strains have a variety of optional RNA editing sites that we report, many of which are unknown to date.

## Introduction

Hepatitis delta virus (HDV) is a subviral agent that uses the envelope of the hepatitis B virus (HBV) to assemble infectious HDV progeny virions. With neither an FDA-approved therapy to treat it [1] nor a dedicated vaccine to prevent it, hepatitis B-hepatitis D (HEPB-HEPD) is the most severe form of chronic hepatitis in humans [2, 3]. It is estimated that a few dozen million people are currently infected by it [4, 5]. However, research of HDV which was recognized as a distinct agent only in 1977 [6, 7] is still at a stage where there is much left to discover.

Bioinformatics methodologies have been successfully utilized in hepatitis virus research [8, 9]. For analyzing RNA folding predictions by energy minimization as was utilized for the present HDV application, several approaches of representing the RNA secondary structure have been devised over the years. Among which are: a full graph representation with nucleotide-node semblance [10], a coarse-grain tree-graph representation with motif-node semblance [11], and a full tree forming a homeomorphically irreducible tree [12]. The Vienna RNA package [13] includes implementation of these representations as well as additional ones. We use the Vienna RNA package for studying conformational switching in HDV, relying first and foremost on the dot-bracket representation (equivalent to a full graph representation) where unpaired nucleotides are represented by dots and paired ones by matching brackets [13, 14]. Conformational switching can be studied by RNA folding prediction of sequences that are contained in databases, be it a riboswitch database such as in [15] or in the case of HDV, the HDV database (HDVdb) [16] that contains a variety of HDV strains.

Combining computational and experimental approaches as part of an examination of specific functions of RNA structure motifs is commonplace in the research of a variety of viruses (e.g. HCV, the hepatitis C virus [17–19]). For example, stem-loop (SL) structures are motifs which are observed in a host of viruses, e.g. [20]. We performed an analysis of SL motifs in viruses in a former work [21]. We then carried out the same idea specifically for HDV [22], where we performed a structural analysis on the 512 genomic sequences present in HDVdb [16] and are from all eight known HDV genotypes [23], using energy minimization predictions and concepts described above such as the coarse-grain tree-graph representation [11]. Additionally, we explored the peculiar RNA editing site discovered in genotype 3 which occurs by a conformational switch [24, 25]. Based on that and on the methodology described in [21, 22], we used an eigenvalue analysis of the Laplacian matrix corresponding to the coarse-grain tree-graph representation of RNA secondary structure applied on HDV strains from HDVdb. An important result of this work was the prediction of the existence of strains which exhibit RNA editing sites not only in genotype 3 of Peru in South America but also in other genotypes, specifically genotype 7 of Cameroon in Africa [22]. Experimental evidence for this prediction was shown in [26]. Observing that the RNA editing site (called amber/W site) responsible for RNA editing in genotype 3 (AUAGU [25]) differs by a single nucleotide from a representative editing site responsible for RNA editing in genotype 7 (AUAGG [26]), in this work, we set off to re-examine the method by which we tried to locate in [22] additional strains in HDVdb that exhibit similar motifs. Hence, we opted for the deployment of a flexible search methodology rather than just the rigid search for the well-known RNA editing site (amber/W) of genotype 3 [25], AUAGU. We used RNAfold from the Vienna RNA package [14] (in principle, same could have been done with mfold/UNAFold [27] and RNAstructure [28]) to isolate the most probable candidate strains in HDVdb. In doing so, we succeeded in locating not only additional candidate strains of interest in genotype 3 but also candidate strains in genotype 7 discovered in HDVdb for the first time. As for other HDV genotypes (1, 2, 4, 5, 6, 8), in the absence of experimental data on editing sites therein, we settled for accumulating information on certain patterns appearing in candidate strains retrieved by our method, which may be of future interest but are not certain as the candidates we report here on genotypes 3 and 7. While this information is out of scope of the present work, it is readily available upon request.

## Materials and methods

The abovementioned observation on two experimentally discovered RNA editing sites differing one from another by a single nucleotide [25, 26] prompted us to consider a new search methodology within HDV strains present in HDVdb. As a starting point, we used the canonical RNA editing site AUAGU [25], which translates to ACTAT after RNA-to-DNA and reverse complement conversions to match the format in which HDV strains are stored in HDVdb. According to [25], we occasionally refer to the RNA editing site as amber/W.

**Figure 1:**
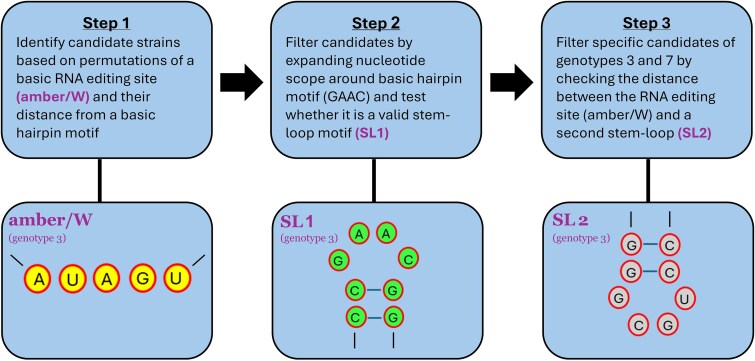
High-level flow of the three-step methodology applied by us in this work on the 512 strains in HDVdb.

A three-step methodology was applied to locate new strains across all 512 HDV strains available in HDVdb (Fig. 1).

### Step 1: sequence-based identification of candidates for the amber/W editing site and SL1

All pentanucleotides with up two substitutions with respect to the base editing site (AUAGU/ACTAT) were identified, exemplified by the AUAGG/CCTAT RNA editing site described in [26]. This means that in total we allowed 106 potential amber/W possibilities (1 base quintet, 15 single-modification quintets, 90 double-modification quintets).

**Figure 2:**
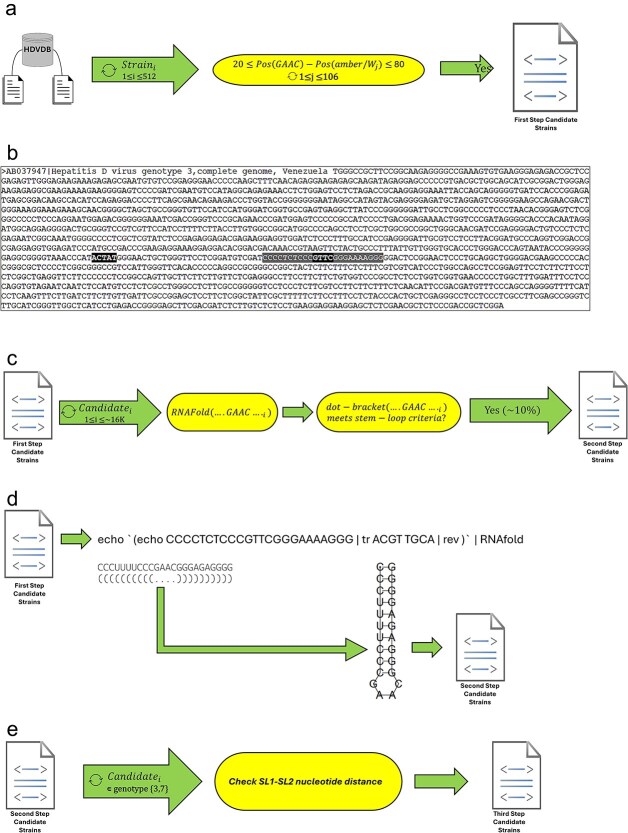
(a) Flow of the first step. Executed on the 512 strains in HDVdb. On each strain 106 possible editing sites are being searched upstream at a distance between 20 and 80 from GTTC, a basic representation of the SL motif SL1. (b) A candidate identified in the first step with the classic amber/W motif ACTAT at distance 44 from SL1, centered at GTTC which was the search target. GTTC is highlighted surrounded by 10 nucleotides on each side. (c) Flow of the second step. Executed on the ~16 K candidate strains identified in the first step. The scope around GTTC, the basic representation of SL1, is widened to 24 nucleotides sent to RNAfold and tested according to SL validity criteria. About 90% of the ~16 K candidates are thus filtered out of the list. (d) Action of the second step over the candidate from (b) resulting in retaining it in the candidates list. (e) Flow of the third step executed on selected candidate strains of genotypes 3 and 7 identified in the second step. Distance between SL1 and SL2 within these candidates is checked to match uniform values (and expected values in the case of genotype 3 where the distance is known).

For each identified pentanucleotide in each strain (where it may appear multiple times), we searched for the nearest GAAC (converted to GTTC as stored in HDVdb), i.e. the minimal unpaired segment of the external loop in the SL structure known as SL1 [25]. We limited the absolute value of the upstream nucleotide distance between amber/W and SL1 to lie between 20 and 80. That is due to the fact that in both discovered RNA editing sites [25, 26], this distance stands on a value of 44 (measured between the starting nucleotide of each motif), yet we wished to allow some widening of the search scope to possibly reveal new patterns, especially in genotypes other than 3 and 7 where no editing site information is yet in existence. The first step is outlined in Fig. 2a and b.

### Step 2: structure-based search for SL1

Candidate sequences obtained in Step 1 were examined for the integrity of the SL motif known as SL1. The sequence scope around the minimal hairpin motif, GAAC/GTTC was widened to include 20 additional nucleotides, 10 on either side. Our goal was to determine whether the sequence under examination forms a valid SL structure or is merely a random nucleotide sequence of no relevance. The resulting 24-nucleotide sequence was used as an input to predict RNA secondary structure using RNAfold [14]. Valid SL structures were selected based on the following criteria: (i) the middle four nucleotides are unpaired, (ii) a minimum of six paired bases appear around the unpaired middle part. The resulting reduced candidate list includes the HDVdb strain identifier, the amber/W sequence motif, and the corresponding distance between this amber/W motif and SL1. The second step is outlined in Fig. 2c and d.

### Step 3: verification of SL2 on strains with experimentally known motifs

At this point, we further examined strains which corresponded with the experimentally known genotypes that exhibit the peculiar RNA editing mechanism by a conformational switch [25, 26], i.e. strains from genotypes 3 and 7 (and possibly from an undefined genotype), with an exact amber/W to SL1 nucleotide distance of 44. On these strains, we conducted an additional verification step where the distance between SL1 and SL2, the second SL motif in [25, 26], was tested as follows. SL2 sequences (CCGCAG for genotype 3 and CCGAGG for genotype 7) were taken from [25] and assisted by [26] for genotype 3 and genotype 7, respectively. SL1 to SL2 distances (488 for genotype 3 and a speculated 316 for genotype 7, allowing <1% variance), taken from [25] for genotype 3 and assisted by [26] for genotype 7, were then applied for the verification of SL2. The third step is outlined in Fig. 2e.

The steps above can also be described by the following pseudo-code that can be assisted along with the figures:

**Algorithm for computational identification and filtering**

***Results*** = {}

***T*** = set of sequences generated from “ACTAT” allowing up to 2 mismatches

For each sequence ***S*** in ***HDVdb***:

***Candidates*** = {}

For each index 0 ≤ ***i*** < len(***S***):

For each sequence ***S’*** in ***T***:

If ***S[i,i+4]*** equals ***S’***:

For each distance 20 ≤ ***d*** ≤ 80:

If ***S[i+4+d, i+4+d+3]*** equals to “GTTC”:

***S”*** = reverse complement of ***S[i+4+d-10, i+4+d+13]***

***R*** = RNAfold(***S”***)

If ***R*** is a stable stem: (explained in Methods)

Add (***i, S’, d, S”, R***) to ***Candidates***

For each ***C*** = (***i, S’, d, S”, R***) in ***Candidates:***

IF no SL2 in ***S:*** (explained in Methods)

Remove ***C*** from ***Candidates***

Find the best candidate ***Best*** in ***Candidates*** using the constraints (explained in Methods)

Add (***S***, ***Best***) to ***Results***

## Results

The application of the first step described above resulted in ~16 000 candidate amber/W-SL1 pairs (bearing in mind that multiple candidates exist per single HDVdb strain due to the flexibility we enabled for amber/W-SL1 distance). Application of the second step disqualified about 90% of the candidates, leaving us with ~1600 candidates for close genotype-based examination. Selection of genotypes 3, 7 and “undefined genotype” reduced the number to 440. Expectedly, the third step applied to relevant genotype 3 and 7 candidates did not change their number. As stated before, in this section, we focus solely on findings from genotypes 3 and 7.

### Genotype 3—Validation

The first criterion by which to verify correctness of our method was validating that the genotype 3 strain which was outlined in [25], the HDVdb identifier of which being L22063, is present in our candidate list. Indeed, it was with the correct amber/W sequence (ACTAT), amber/W-SL1 distance (44), and SL1 both in FASTA and in dot-bracket representations (as in Fig. 2b in [25]). Table 1 demonstrates the verification of strain L22063 of genotype 3 as it appears in the main candidate list obtained from running our abovementioned three steps.

**Table 1:** Strain L22063 on our list of candidates.

**Amber/W**	**Genotype**	**HDVDB ID**	**SL1-Amber/W distance**	**Hairpin loop**	**Hairpin loop dot-bracket**
ACTAT	3	L22063	44	CCCTTTTCCCGAACGGGAGAGGGG	(((((((((( ….))))))))))

### Genotype 3—further verification and new findings

In an analysis of the rest of the genotype 3 candidates, 10 additional candidate strains were found. Eight of which parallel to L22063 in the sense they contain a similar amber/W sequence of ACTAT/AUAGU at a similar nucleotide distance (44) from SL1. This is a corroboration of a finding we first demonstrated in [29]. The other two are new candidates with a different amber/W of ACCAT/AUGGU outlined here for the first time. Table 2 demonstrates the candidates of genotype 3 from HDVdb. A detailed list of candidates from genotype 3 is downloadable from the resource specified in the Data Availability section.

**Table 2:** Genotype 3 candidate strains: Casey’s ACTAT L22063, other ACTAT strains, and new ACCAT strains.

Amber/W	HDVDB ID	SL1-Amber/W distance	Hairpin loop dot-bracket
ACTAT	L22063	44	(((((((((( ….))))))))))
ACTAT	HW649778	44	(((((((((( ….))))))))))
ACTAT	HF679404	44	(((((((((( ….))))))))))
ACCAT	LT604954	44	(((((((((( ….))))))))))
ACTAT	HF679405	44	(((((((((( ….))))))))))
ACCAT	LT604955	44	(((.(((((( ….)))))).)))
ACTAT	AB037949	44	(((((((((( ….))))))))))
ACTAT	AB037948	44	(((((((((( ….))))))))))
ACTAT	HF679406	44	(((((((((( ….))))))))))
ACTAT	KC590319	44	(((((((((( ….))))))))))
ACTAT	AB037947	44	(((((((((( ….))))))))))

### Genotype 7—new findings

**Figure 3:**
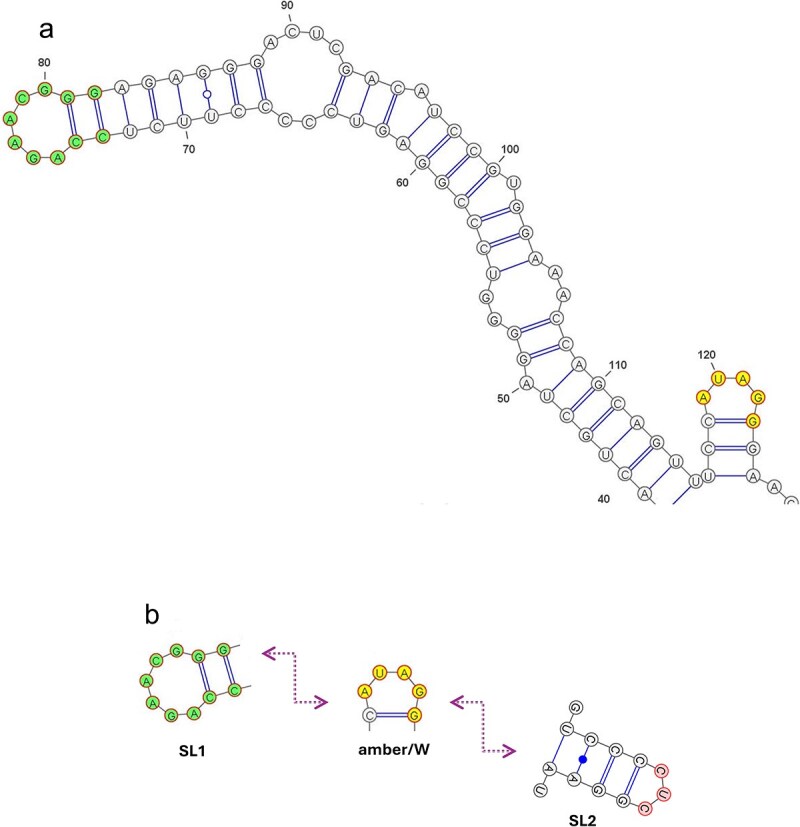
(a) Folding prediction by energy minimization of SL1 and amber/W in the strain KM110802 from HDVdb, folded by RNAfold [14] and plotted by VARNA [32]. This strain was discovered by our work as one of the new candidates of HDV genotype 7 with an amber/W motif AUAGG coupled to an SL1 motif. (b) Schematic RNA secondary structure motifs in strain KM110802 from HDVdb as folded by RNAfold and plotted by VARNA, with SL2 added to SL1 and amber/W that are fully shown in (a) with an accurate folding prediction by energy minimization. SL2 is relatively far away from SL1 and amber/W (the nucleotide distance between SL1 and SL2 is 316) and therefore SL2 is not shown in (a), as the folding prediction by energy minimization of the entire sequence that includes SL1, amber/W, and SL2 becomes inaccurate.

Analysis of genotype 7 candidates produced new findings of high interest, the ones we sought after. 13 strains demonstrated striking similarity to the motif properties shown in [26]. It is noteworthy to point out that strain dFr1024 of [26] is not present in HDVdb. Two of the 13 contain CCTAT/AUAGG, the exact RNA editing site of [26], as well as similar SL1 and SL2 motifs as retrieved from fig. 4 of [26]. The other 11 contain a slightly modified quintet, CCCAT/AUGGG, which may prove to be an alternative RNA editing site. Table 3 demonstrates the newly found candidates of genotype 7 from HDVdb. Figure 3a and b depicts the secondary structure of one of the strains in Table 3, namely KM110802, showing its amber/W in yellow and SL1 motif in green. A detailed list of candidates from genotype 7 is downloadable from the resource specified in the Data Availability section.

**Table 3:** Genotype 7 candidate strains—new CCCAT strains and CCTAT strains.

Amber/W	HDVDB ID	SL1-Amber/W distance	Hairpin loop dot-bracket
CCCAT	AM183333	44	((((((((( …..))))))))).
CCCAT	LT604970	44	(((((((((.. …))))))))).
CCCAT	LT604971	44	.(((((((( … …)))))))).
CCCAT	MG711795	44	.((((((((. …..)))))))).
CCCAT	LT604972	44	.((.((((( … …))))).)).
CCCAT	MG711758	44	((((((((( …..))))))))).
CCCAT	LT594487	44	.(((((((( … …)))))))).
CCCAT	MG711790	44	.(((((((( … …)))))))).
CCCAT	MG711799	44	(((.(((((.. ….))))).)))
CCCAT	MG711791	44	((((((.(( …..)).)))))).
CCCAT	MG711714	44	.((.((((( … …))))).)).
CCTAT	KM110802	44	.(((((((( … …)))))))).
CCTAT	KM110804	44	(((.(((( ….. …)))).)))

### Undefined genotype—new findings

HDVdb also contains strains of an “undefined genotype.” Going through these strains, we were able to locate two strains similar to genotype 3 ACTAT strains of [25] which were also pointed out in [29] as well as three new strains similar to genotype 7 CCTAT strains of [26]. It is safe to assume that the presence of these strains in our final candidate list confirms that their true genotypes are 3 and 7, respectively. We hereby add them to the list of findings of importance. Table 4 demonstrates the candidates of undefined genotype from HDVdb. A detailed list of candidates from undefined genotypes is downloadable from the resource specified in the Data Availability section.

**Table 4:** Undefined genotype candidate strains: Genotype 3 ACTAT strains and genotype 7 CCTAT strains.

Amber/W	HDVDB ID	SL1-Amber/W distance	Hairpin loop dot-bracket
ACTAT	JC493892	44	((((((((((....))))))))))
ACTAT	MA997215	44	((((((((((....))))))))))
CCTAT	AX741144	44	.((((((((......)))))))).
CCTAT	JA417541	44	.((((((((......)))))))).
CCTAT	MA997223	44	.((((((((......)))))))).

### Other genotypes

In the analysis of the remaining six genotypes (i.e. 1, 2, 4, 5, 6, 8), we faced an inherent stumbling block whereas there is no *in vitro* information to rely upon, especially when looking for an RNA editing site. Via the abovementioned motif distance flexibility, which we enabled in our search method, in genotypes 1 and 4, we were able to find preserved motifs at a nucleotide distance other than 44. Namely, a possible RNA editing site GCTAG at a nucleotide distance of 66 from SL1. We hope that this could serve as a seed for future *in vitro* studies. Samples of these findings appear, with due caution, in Table 5. The full list of candidates from genotypes 1 and 4 is downloadable from the resource specified in the Data Availability section.

**Table 5:** Sample of candidates from genotypes 1 and 4.

Possible editing site	Genotype	HDVDB ID	Motif distance	Hairpin loop dot-bracket
GCTAG	1	MG711668	66	.(((((((. … …))).)))).
GCTAG	4	AB118818	66	.((((.(((. …..))).)))).

## Discussion and conclusion

The new findings described herein bring forth a host of publicly available candidate strains for further research both the strains from HDVdb [16] with known RNA editing sites and the ones with previously unknown RNA editing sites. A subsequent structural analysis, experimentally and also computationally in 3D (e.g. [30, 31]), is now possible for performing with these strains.

Based on our recent findings in [22], in which by using an eigenvalue mathematical analysis, we identified that the peculiar RNA editing mechanism by a conformational switch from an unbranched to a branched structure that was known to exist in HDV genotype 3 [25] also exists in HDV genotype 7, herein we devised and applied a search methodology to identify all strains present in HDVdb that exhibit this peculiar RNA editing mechanism. This approach resulted both in corroborating existing knowledge in the field (for genotype 3) and in discovering candidate strains with modified RNA editing sites (for genotype 7). We disseminate our findings for further analyses by providing the special strains in Tables 2–5 found in HDVdb (Fig. 3 is an illustrative example of the secondary structure drawing of one of the strains from genotype 7, highlighting its amber/W and SL1 motif) with the aim of understanding better the underlying biological mechanism of RNA editing in HDV.

Once further biological experiments are carried out, future work is set to include widening of the search methodology to enable flexibility not only in the sequence of the RNA editing site but also in the stem loops. Findings in other genotypes such as 1 and 4 for which we offer candidate strains in this paper will enable us to meaningfully enlarge the scope of data under test, also exploring databases other than HDVdb such as vHDvDB 2.0 and NCBI Virus.

**Key Points**

- Our results significantly widen the research scope of the RNA editing mechanism by a conformational switch from an unbranched to a branched structure in hepatitis delta virus.
- A variety of strains from HDV database can be further selected for experimental study, especially in hepatitis delta virus genotype 7.
- The bioinformatics methods that are described in this paper can be further applied toward discovering other RNA functional elements in new locations.

## Abbreviations

AWK, Aho, Weinberger, Kernighan scripting language

DNA, deoxyribonucleic acid

FDA, (U.S.) Food and Drug Administration

HEPB, hepatitis B

HEPD, hepatitis D

HCV, hepatitis C virus

HDV, hepatitis D virus

HDVdb, hepatitis D virus database

RNA, ribonucleic acid

SL, stem loop

## Data Availability

All data and software described in the study are provided at: https://github.com/comp-bio-hdv/Data-Availability.git. The software was implemented as a suite of AWK and Linux shell scripts. For ease of search, convenience of referral and data integrity, we split HDVdb genomes into one file per genome (i.e. 512 files) and one line per file. Follow the installation and execution process as described in the file *README.pdf.*
